# Association Between Middle Mesial Canals and Additional Distal Root Canal Anatomy in Mandibular First Molars: A Cone‐Beam Computed Tomography Study in an Iranian Population

**DOI:** 10.1155/ijod/1262780

**Published:** 2026-10-11

**Authors:** Tooba Aslanabadi, Tahmineh Razi, Saeedeh Galledar, Naghmeh Rahimi Darehchi, Parisa Jalali, Saeed Rahimi

**Affiliations:** ^1^ Dentist Private Practice Tabriz Iran; ^2^ Department of Radiology Faculty of Dentistry Tabriz University of Medical Sciences Tabriz Iran tbzmed.ac.ir; ^3^ Department of Endodontics Faculty of Dentistry Ardabil University of Medical Sciences Ardabil Iran arums.ac.ir; ^4^ Dentist Private Practice Budapest Hungary; ^5^ Department of Endodontics Faculty of Dentistry Tabriz University of Medical Sciences Tabriz Iran tbzmed.ac.ir; ^6^ Student Research Committee Tabriz University of Medical Sciences Tabriz Iran tbzmed.ac.ir; ^7^ Department of Endodontics Faculty of Dentistry Dental and Periodontal Research Center Tabriz University of Medical Sciences Tabriz Iran tbzmed.ac.ir

**Keywords:** additional distal canal, cone-beam computed tomography, mandibular first molar, middle mesial canal, radix entomolaris, root canal morphology

## Abstract

**Background:**

A comprehensive understanding of root canal morphology is essential for successful endodontic treatment. Anatomical variations such as middle mesial canals (MMCs), second distal canals, and additional distal roots may complicate canal identification and instrumentation. Although the prevalence of these anatomical features has been investigated in different populations, evidence regarding their potential coexistence within the same tooth remains limited. This study evaluated the prevalence of MMCs and their association with second distal canals and additional distal roots in mandibular first molars using cone‐beam computed tomography (CBCT) in an Iranian population.

**Methods:**

This retrospective cross‐sectional study analyzed 200 CBCT examinations obtained from patients referred to the Department of Oral and Maxillofacial Radiology, Tabriz University of Medical Sciences, between 2019 and 2021. The prevalence of MMCs, second distal canals, and additional distal roots was recorded. Associations among anatomical variables were evaluated using chi‐square tests and multivariable logistic regression analyses adjusted for age and sex.

**Results:**

MMCs were identified in 26 of 200 mandibular first molars (13.0%), while second distal canals were observed in 64 cases (32.0%). Among teeth with a second distal canal, 15 cases (23.4%) also exhibited an additional distal root. In multivariable analysis, the presence of an MMC was associated with higher odds of a second distal canal (adjusted odds ratio [OR] = 2.62; 95% confidence interval [CI]: 1.11–6.20; *p* = 0.027). Among teeth with a second distal canal, an association was also observed between MMC presence and additional distal roots (adjusted OR = 6.00; 95% CI: 1.33–27.16; *p* = 0.020). However, the relatively small number of positive cases, particularly for additional distal roots, resulted in wide CIs and limited the precision of these effect estimates.

**Conclusions:**

Within the limitations of this retrospective CBCT‐based study, MMCs were associated with the presence of additional distal canal anatomy in mandibular first molars. The findings suggest a possible relationship between mesial and distal anatomical complexity; however, the relatively small number of positive cases and wide CIs warrant cautious interpretation. These associations should be considered exploratory rather than definitive and require confirmation in larger, multicenter studies with greater statistical precision.

## 1. Introduction

Successful endodontic treatment relies on thorough identification, cleaning, shaping, disinfection, and obturation of the entire root canal system. Failure to detect anatomical variations remains a major cause of endodontic treatment failure, as untreated canals may harbor microorganisms and necrotic tissue that sustain persistent periapical inflammation despite technically acceptable treatment of the identified canals [1–3]. Consequently, comprehensive knowledge of root and canal anatomy is considered a fundamental prerequisite for predictable endodontic therapy and long‐term tooth preservation [1, 4, 5].

Among permanent teeth, mandibular first molars are among the most frequently subjected to root canal treatment because of their early eruption, prolonged exposure to the oral environment, and increased susceptibility to caries and pulpal disease [4]. These teeth are characterized by considerable anatomical complexity and exhibit substantial variations in root and canal morphology. Although the conventional anatomy of mandibular first molars consists of two roots, with two canals in the mesial root and one or two canals in the distal root, numerous deviations from this pattern have been documented. These include middle mesial canals (MMCs), additional distal canals, radix entomolaris, radix paramolaris, canal isthmuses, canal bifurcations, and complex canal merging patterns [4, 6–10]. Failure to identify such anatomical structures may compromise canal debridement and adversely affect treatment outcomes [2, 3].

Among these anatomical variations, the MMC has attracted considerable clinical and scientific interest. Initially described by Pomeranz et al. [11], the MMC is located between the mesiobuccal and mesiolingual canals and may occur as an independent canal, a confluent canal, or a narrow communication within the mesial root canal system. The reported prevalence of MMCs varies considerably among studies, ranging from less than 1% to over 20%, reflecting differences in ethnicity, age distribution, diagnostic methodology, and imaging techniques [12–19]. A recent systematic review and meta‐analysis of cone‐beam computed tomography (CBCT) studies demonstrated substantial global variation in MMC prevalence and highlighted the importance of population‐specific investigations when evaluating root canal morphology [19]. Furthermore, a recent CBCT investigation involving 898 mandibular first molars reported a prevalence of 9.6% and showed that most MMCs exhibited confluent configurations, emphasizing their clinical relevance and potential for being overlooked during treatment [20]. These findings indicate that MMCs represent an important source of anatomical complexity that requires careful clinical assessment.

Advances in diagnostic technology have significantly improved the detection of complex root canal anatomy. In particular, CBCT has emerged as a valuable imaging modality for evaluating root canal morphology in vivo. Unlike conventional two‐dimensional radiography, CBCT provides three‐dimensional visualization of dental structures and allows an accurate assessment of anatomical variations from multiple perspectives [21, 22]. Numerous studies have demonstrated that CBCT substantially improves the detection of MMCs and other complex canal configurations that may remain undetected on conventional radiographs [10, 15–22]. Recent CBCT‐based investigations have further refined our understanding of MMC morphology and spatial relationships within the mesial root, facilitating more accurate anatomical characterization and treatment planning [18–20]. Nevertheless, despite its advantages, CBCT remains limited by image artifacts and lower spatial resolution compared with micro‐computed tomography, which continues to be regarded as the reference standard for ex vivo anatomical investigations [14, 23].

In addition to mesial root variations, the distal root of the mandibular first molar exhibits substantial anatomical diversity. Previous studies have documented the presence of a second distal canal, complex distal canal systems, and supernumerary roots such as radix entomolaris [6–8]. The prevalence of these anatomical variations differs markedly among ethnic populations. Higher frequencies of additional roots and canals have generally been reported in East Asian populations compared with European, Middle Eastern, and African populations [8, 24, 25]. Such findings suggest that genetic and developmental factors may contribute to population‐specific patterns of root canal morphology [10, 25]. Consequently, investigations conducted within specific populations remain essential for improving clinicians’ awareness of local anatomical characteristics and reducing the risk of missed canals during treatment.

The developmental basis of the root canal morphology may provide important insights into the coexistence of multiple anatomical variations within the same tooth. Root development is governed by complex interactions among genetic, molecular, and environmental factors during odontogenesis. Hertwig’s epithelial root sheath plays a critical role in determining root number, root form, and canal configuration by regulating root dentin formation and root bifurcation patterns [26–28]. Minor disturbances during these developmental processes may result in accessory canals, additional roots, canal bifurcations, and other morphological variations. Therefore, anatomical variations observed in different regions of the same tooth may not represent isolated events but rather manifestations of a broader developmental tendency toward increased anatomical complexity.

This developmental perspective raises an important yet insufficiently investigated question: whether the presence of one anatomical variation may be associated with the occurrence of additional variations elsewhere within the same tooth. Specifically, the presence of an MMC may indicate a greater likelihood of complex distal root anatomy, including second distal canals or additional distal roots. Although numerous studies have evaluated the prevalence of MMCs and distal anatomical variations separately, relatively few investigations have explored possible associations among these structures [14, 19, 20, 25, 29]. As a result, the biological and developmental relationships between mesial and distal root anatomical complexity remain poorly understood.

The existing literature has predominantly focused on prevalence estimates rather than anatomical interrelationships. Most CBCT‐based studies have reported MMCs, distal canals, and additional roots as isolated anatomical findings without examining whether these variations tend to coexist within the same tooth [10, 14, 19, 20, 25, 29]. Furthermore, studies evaluating such associations have often been limited by modest sample sizes, methodological heterogeneity, and population‐specific characteristics. Consequently, a significant gap remains in our understanding of the developmental and clinical interconnections among anatomical variations within the mandibular first molars.

From a clinical perspective, investigating these associations may provide benefits beyond simple prevalence reporting. Awareness of potential anatomical correlations may encourage clinicians to perform a more comprehensive exploration of the pulp chamber floor and root canal system when a particular variation is detected. Such awareness could improve diagnostic vigilance and reduce the risk of missed canals during endodontic treatment [2, 3, 12, 13, 21, 22]. However, anatomical associations should not be interpreted as deterministic predictors; rather, they may serve as indicators of increased anatomical complexity, requiring additional investigation.

The Iranian population represents a particularly relevant group for studying root canal anatomy because of its considerable ethnic diversity and unique geographic position at the crossroads of multiple historical migration routes. Previous investigations conducted in various Iranian populations have reported prevalence rates for MMCs and other anatomical variations that differ from those observed in neighboring and distant populations [15–18, 30–32]. Notably, a recent CBCT study conducted in a Persian population reported a prevalence of MMCs comparable to that observed in several Asian populations, further supporting the existence of population‐specific anatomical characteristics [18]. Despite these findings, evidence regarding the relationship between MMCs and distal anatomical variations in the Iranian mandibular first molars remains scarce.

Therefore, the present study aimed to evaluate the prevalence of MMCs, second distal canals, and additional distal roots in mandibular first molars using CBCT in an Iranian population. Furthermore, the study sought to investigate the association between MMCs and distal anatomical variations, thereby contributing to a more comprehensive understanding of the root canal morphology and its developmental complexity. By exploring these relationships in a well‐defined CBCT dataset, the present study seeks to provide clinically relevant information while expanding current knowledge regarding anatomical interconnections within mandibular first molars.

## 2. Materials and Methods

### 2.1. Study Design and Ethical Approval

This retrospective cross‐sectional observational study was conducted to investigate the association between MMCs, second distal canals, and additional distal roots in mandibular first molars using CBCT. The study protocol was approved by the Ethics Committee of Tabriz University of Medical Sciences (Approval Number IR.TBZMED.REC.1401.525) and was performed in accordance with the Declaration of Helsinki. Because previously acquired CBCT images were retrospectively analyzed, informed consent requirements were waived by the institutional review board.

The study was designed and reported in accordance with the Preferred Reporting Items for Epidemiologic Cross‐sectional Studies on Root and Root Canal Anatomy Using Cone‐beam Computed Tomographic Technology recommendations to ensure methodological transparency and reproducibility.

### 2.2. Study Population and Sample Selection

The study population consisted of patients referred to the Department of Oral and Maxillofacial Radiology, Tabriz University of Medical Sciences, for CBCT imaging between 2019 and 2021. Consecutive CBCT examinations meeting the eligibility criteria were screened for inclusion to minimize selection bias.

CBCT scans were included when they fulfilled the following criteria:

1. The presence of at least one fully erupted permanent mandibular first molar.
2. Complete root development with closed apices.
3. Adequate image quality for anatomical evaluation.
4. The absence of significant image artifacts affecting visualization of the root canal system.

Scans were excluded if any of the following conditions were present:

1. Previous endodontic treatment.
2. Extensive coronal restorations producing image distortion.
3. Internal or external root resorption.
4. Root fractures.
5. Periapical lesions causing severe anatomical distortion.
6. Developmental anomalies affecting tooth morphology.
7. Inadequate image quality.

After the application of the eligibility criteria, 200 CBCT examinations were included in the final analysis.

All included CBCT examinations contained complete information for the variables of interest, including age, sex, tooth side, and anatomical findings. Cases with incomplete demographic information or inadequate image quality were excluded during the eligibility assessment. Therefore, no missing data were present in the final dataset, and no imputation or other missing‐data handling procedures were required.

### 2.3. Sample Size Considerations

Because of the retrospective nature of the study, all eligible CBCT examinations available during the study period were included. No formal a priori sample size calculation was performed. Nevertheless, the entire accessible dataset meeting the inclusion criteria was analyzed to maximize statistical power and improve the precision of the prevalence estimates.

Considering the relatively low prevalence of MMCs and additional distal roots reported in previous studies, the findings related to uncommon anatomical variants should be interpreted with appropriate caution. Confidence intervals (CIs) were therefore reported to reflect the precision of the estimated associations.

To avoid dependency between the bilateral observations, only one mandibular first molar from each patient was included in the study. Therefore, the patient and tooth units were considered equivalent for the purpose of the analysis, and each observation was treated as statistically independent.

### 2.4. CBCT Acquisition Protocol

All CBCT scans were obtained using a standardized imaging protocol with a Newtom VGi device (Verona, Italy) using a conical X‐ray beam and a flat panel detector with a resolution of 1536 × 1920 pixels and exposure parameters of 110 kVp.

The patients’ images were reviewed by a single observer on a 19‐inch Philips (190B) desktop computer with a liquid crystal display (LCD) screen, a resolution of 1208 × 1024, and a 32‐bit color depth. The images were viewed in a semi‐dark room. Following standardization of head orientation in the CBCT software, the axial slice thickness was set at 0.3 mm.

Images were reconstructed and evaluated using NNT Viewer software (Version 2.21, NewTom, Verona, Italy) under standardized viewing conditions. Brightness, contrast, and magnification adjustments were permitted when necessary to optimize the visualization of root canal anatomy (Figures 1 and 2).

**Figure 1:**
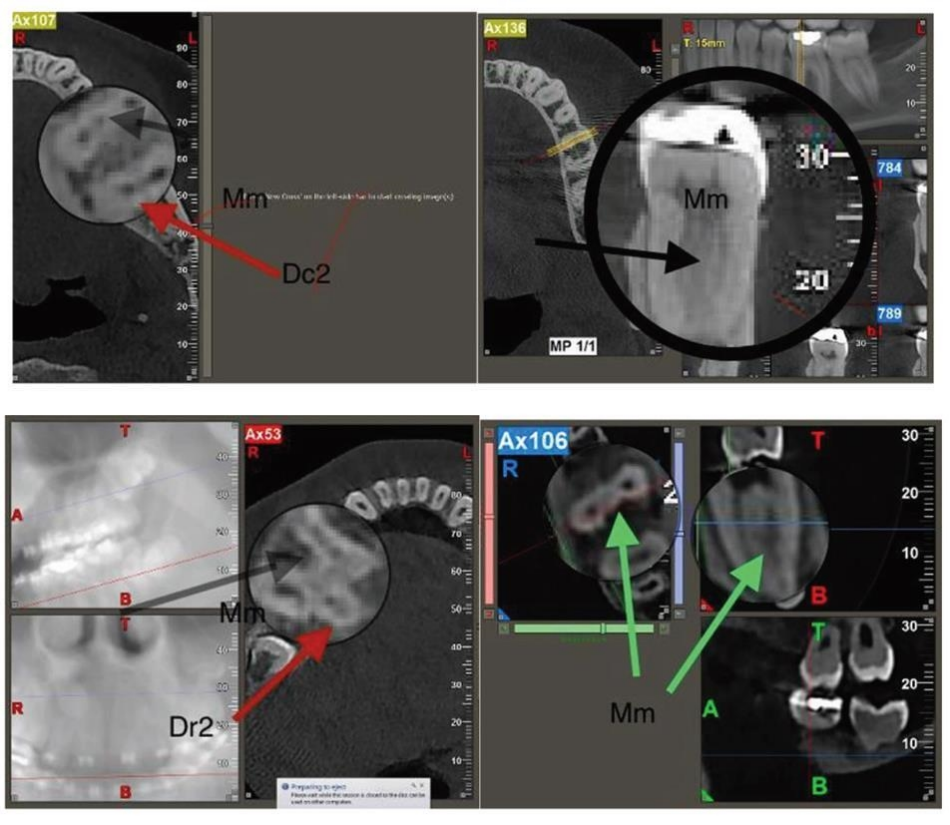
CBCT images of the investigated medial canal (Mm), second distal root (Dr2), and second distal canal (Dc2) are shown.

**Figure 2:**
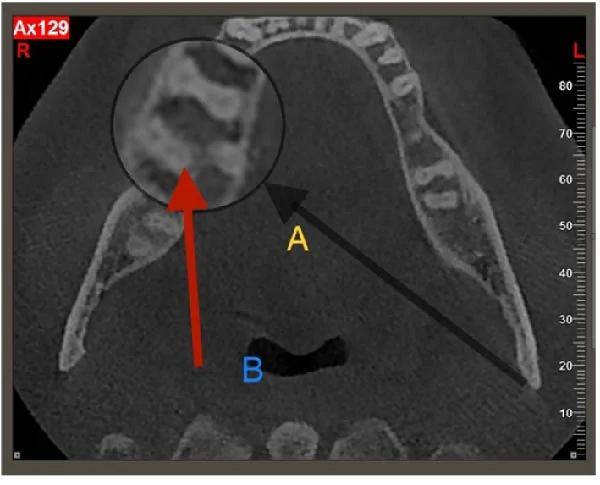
CBCT image of the first molar of the lower jaw, which has five canals. The second distal root “A” and the second root of distal canal “B” are shown.

### 2.5. Image Evaluation Procedure

All images were independently evaluated by two experienced examiners, including an oral and maxillofacial radiologist and an endodontist, both with substantial experience in CBCT interpretation.

The evaluation was performed using multiplanar reconstruction views, including axial, coronal, and sagittal sections. Sequential image slices were assessed from the pulp chamber floor to the root apex to ensure a comprehensive examination of root canal morphology.

When disagreement occurred between the examiners, a joint reassessment session was conducted. If consensus could not be achieved, a third senior endodontist was consulted, and the final decision was reached through discussion.

### 2.6. Examiner Calibration and Reliability Assessment

Prior to the main evaluation, both examiners participated in a calibration exercise involving 30 randomly selected CBCT scans that were not included in the final study sample.

The calibration process focused on the following:

Identification of MMCs.
Identification of second distal canals.
Identification of additional distal roots.
Standardized interpretation of complex canal configurations.

After a 2‐week interval, the same scans were reevaluated to assess intraobserver reliability.

Interobserver agreement was evaluated using the Cohen kappa coefficient and demonstrated excellent agreement (*κ* = 0.85). Intraobserver agreement values were also calculated and exceeded 0.80 for all assessed anatomical variables, indicating substantial to excellent reproducibility.

### 2.7. Diagnostic Criteria

#### 2.7.1. MMC

A MMC was defined as a distinct canal space located between the mesiobuccal and mesiolingual canals of the mesial root and identifiable on at least two consecutive CBCT slices.

Only clearly distinguishable canal lumens were classified as MMCs. Canal fins, isthmuses, and nonseparable radiolucent communication were not classified as independent MMCs.

### 2.8. Second Distal Canal

A second distal canal was defined as a distinct and clearly identifiable canal lumen within the distal root that could be differentiated from the primary distal canal on at least two consecutive CBCT slices. This criterion was applied to the identifiable canal pathway rather than to the presence of separate canal orifices alone. A separate orifice was not considered sufficient for classification as a second distal canal unless a distinct secondary canal lumen was demonstrated on at least two consecutive slices. Both independent and confluent canal configurations were recorded when a distinct secondary canal pathway was identifiable. Canal bifurcations, fins, isthmuses, or nonseparable radiolucent communication without a clearly distinguishable secondary canal lumen were not classified as a second distal canal.

### 2.9. Anatomical Classification Framework

The root and root canal configurations were interpreted within the internationally recognized classification system proposed by Ahmed et al. [9] for root and root canal morphology. This classification framework was used to standardize the description of additional canal and root configurations and to facilitate comparison with previous anatomical studies. The operational CBCT criteria described above were retained to determine whether a canal or root was sufficiently distinct to be included in the present analysis.

### 2.10. Additional Distal Root

An additional distal root (radix entomolaris) was defined as a completely separate root structure, exhibiting an independent periodontal ligament space and distinct root morphology.

Roots demonstrating only canal bifurcation without clear root separation were not classified as additional roots.

### 2.11. Variables Recorded

The following variables were collected for each eligible mandibular first molar:

• Presence or absence of MMC.
• Presence or absence of a second distal canal.
• Presence or absence of an additional distal root.
• Patient sex.
• Patient age.
• Tooth side (right or left).

### 2.12. Statistical Analysis

Statistical analyses were performed using SPSS software (Version 22; IBM Corp., Armonk, NY, USA).

Descriptive statistics were used to summarize the prevalence data. Categorical variables were presented as frequencies and percentages, whereas continuous variables were summarized using means and standard deviations.

Associations between categorical variables were evaluated using the chi‐square test or Fisher’s exact test, when appropriate.

To investigate potential associations between MMCs and distal anatomical variations, multivariable logistic regression analyses were performed. Odds ratios (ORs) and corresponding 95% CIs were calculated.

Age and sex were entered as covariates in the regression models to reduce potential confounding effects.

Because bilateral mandibular first molars may not represent completely independent observations, prevalence estimates and regression findings were interpreted cautiously. Statistical significance was established at *p*  < 0.05.

The results of the logistic regression analyses were interpreted as measures of association rather than prediction or causality because of the cross‐sectional nature of the study.

Subgroup analyses were performed according to sex and tooth side using the chi‐square test or Fisher’s exact test, as appropriate. Age and sex were included as covariates in the multivariable logistic regression models to adjust for potential confounding. Formal interaction analyses (e.g., inclusion of interaction terms between the MMC status and demographic variables) were not conducted because of the limited number of positive cases and insufficient statistical power to reliably evaluate interaction effects. No sensitivity analyses were performed because the final dataset was complete and consisted of all eligible CBCT examinations meeting the predefined inclusion criteria.

## 3. Results

### 3.1. Study Population

A total of 200 CBCT examinations that fulfilled the eligibility criteria were included in the final analysis. The study population consisted of 123 males and 77 females with a mean age of 27.85 ± 13.96 years (range: 18–68 years). All included scans demonstrated sufficient image quality for anatomical assessment and were successfully evaluated by both examiners (Figure 3).

**Figure 3:**
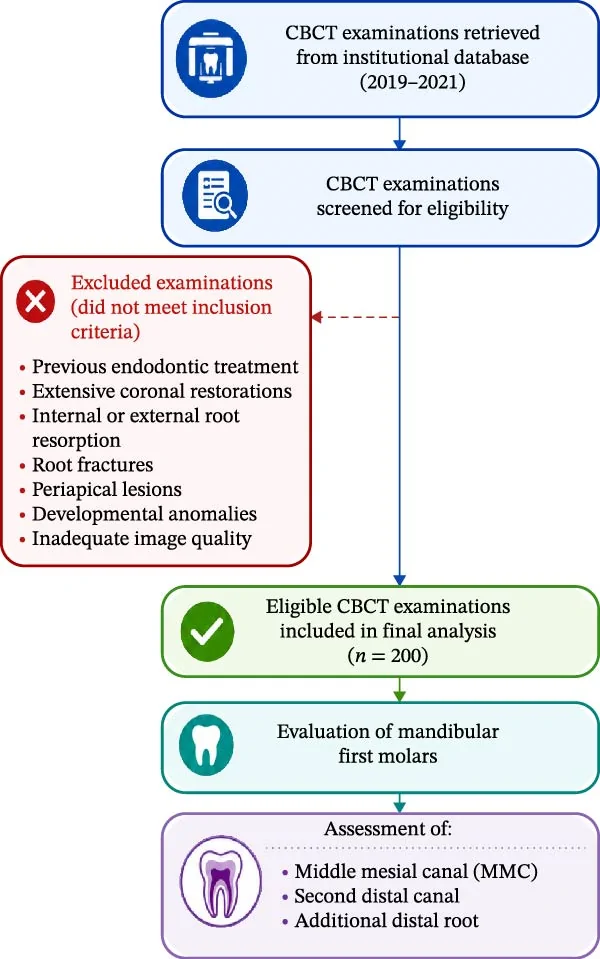
Flow diagram illustrating the selection process of CBCT examinations and assessment of mandibular first molars included in the study.

Complete data were available for all included CBCT examinations. No missing data were identified for any of the variables of interest, including age, sex, tooth side, MMC, second distal canal, or additional distal root. Consequently, all 200 eligible CBCT examinations were included in the statistical analyses.

### 3.2. Prevalence of MMCs

MMCs were identified in 26 of the 200 evaluated mandibular first molars, corresponding to an overall prevalence of 13.0% (95% CI: 8.8%–18.7%).

The distribution of MMCs according to sex and tooth side is presented in Table 1. Although MMCs appeared slightly more frequently in females than in males, the difference did not reach statistical significance (*p* > 0.05). Similarly, no significant difference was observed between right and left mandibular first molars (*p* > 0.05).

**Table 1:** Distribution of middle mesial canals, second distal canals, and additional distal roots according to sex and tooth side.

Anatomical variation	Sex	Not present, *n* (%)	Left, *n* (%)	Right, *n* (%)	Bilateral, *n* (%)	*p*‐Value
Middle mesial canal	Female	67 (87.0)	4 (5.2)	4 (5.2)	2 (2.6)	0.990
	Male	107 (87.0)	7 (5.7)	6 (4.9)	3 (2.4)	—
Second distal canal	Female	42 (54.5)	6 (7.8)	13 (16.9)	16 (20.8)	0.174
	Male	94 (76.4)	5 (4.1)	11 (8.9)	13 (10.6)	—
Additional distal root	Female	30 (85.7)	1 (2.9)	1 (2.9)	3 (8.6)	0.059
	Male	19 (65.5)	1 (3.4)	6 (20.7)	3 (10.3)	—

*Note:* Additional distal root was assessed among mandibular first molars exhibiting a second distal canal.

Abbreviations: CBCT, cone‐beam computed tomography; MMC, middle mesial canal.

### 3.3. Prevalence of Second Distal Canals

A second distal canal was detected in 64 of the 200 mandibular first molars, yielding a prevalence of 32.0% (95% CI: 25.9%–38.9%).

Females demonstrated a numerically higher prevalence of second distal canals than males; however, this difference was not statistically significant (*p*  > 0.05). Likewise, no significant association was observed between the tooth side and the presence of a second distal canal (Table 1).

### 3.4. Prevalence of Additional Distal Roots

An additional distal root (radix entomolaris) was identified in 15 mandibular first molars, corresponding to a prevalence of 7.5% (95% CI: 4.6%–12.0%).

No statistically significant associations were observed between the presence of an additional distal root and patient sex, age group, or tooth side (*p*  > 0.05).

### 3.5. Association Between MMCs and Second Distal Canals

A statistically significant association was observed between the presence of an MMC and the occurrence of a second distal canal.

Among teeth presenting with an MMC, the frequency of a second distal canal was significantly greater than that among teeth without an MMC. Multivariable logistic regression analysis demonstrated that teeth exhibiting an MMC had ~2.6‐fold higher odds of presenting a second distal canal compared with teeth without an MMC (adjusted OR = 2.62; 95% CI: 1.11–6.20; *p* = 0.027) (Tables 2 and 3).

**Table 2:** Association between middle mesial canal and second distal canal/second distal root.

Mid‐mesial canal	Second distal canal: No	Second distal canal: Yes	*p*‐Value
Mid‐mesial canal: No	124 (71.3%)	50 (28.7%)	0.01
Mid‐mesial canal: Yes	12 (46.2%)	14 (53.8%)	—

	Second distal root: No	Second distal root: Yes	*p*‐Value
Mid‐mesial canal: No (among those with 2nd distal canal)	42 (84.0%)	8 (16.0%)	0.014
Mid‐mesial canal: Yes (among those with 2nd distal canal)	7 (50.0%)	7 (50.0%)	—

**Table 3:** Logistic regression for presence of second distal canal (adjusted for age and gender).

Variable	Adjusted OR	95% CI	*p*‐Value
Middle mesial canal (present vs. absent)	2.62	(1.11–6.20)	0.027
Age (per year increase)	0.99	(0.98–1.02)	0.948
Gender (male vs. female)	1.42	(0.75–2.69)	0.278

After adjustment for age and sex, the association remained statistically significant, suggesting that the coexistence of MMCs and second distal canals may reflect increased overall anatomical complexity within the mandibular first molars.

### 3.6. Association Between MMCs and Additional Distal Roots

The relationship between MMCs and additional distal roots was also evaluated.

Among teeth exhibiting a second distal canal, the presence of an MMC was associated with a significantly greater likelihood of an additional distal root. Logistic regression analysis revealed that teeth with MMCs demonstrated approximately six‐fold higher odds of presenting an additional distal root compared with teeth without MMCs (adjusted OR = 6.00; 95% CI: 1.33–27.16; *p* = 0.020) (Table 4).

**Table 4:** Logistic regression for presence of second distal root among patients with second distal canal (adjusted for age and gender).

Variable	Adjusted OR	95% CI	*p*‐Value
Middle mesial canal (present vs. absent)	6.00	(1.33–27.16)	0.020
Age (per year increase)	0.96	(0.90–1.01)	0.141
Gender (male vs. female)	3.62	(0.92–14.30)	0.066

However, the relatively wide CI reflects the limited number of positive cases and indicates the reduced precision of the estimate. Therefore, this finding should be interpreted cautiously.

### 3.7. Multivariable Regression Analysis

Multivariable logistic regression models were constructed to investigate the independent association between MMCs and distal anatomical variations while controlling for age and sex.

The presence of an MMC remained a significant predictor of a second distal canal after adjustment for demographic variables (adjusted OR = 2.62; 95% CI: 1.11–6.20; *p* = 0.027). Likewise, MMC presence remained significantly associated with additional distal roots among teeth exhibiting distal canal complexity (adjusted OR = 6.00; 95% CI: 1.33–27.16; *p* = 0.020).

Neither age nor sex demonstrated a statistically significant independent association with the occurrence of MMCs, second distal canals, or additional distal roots in the adjusted models (all *p* > 0.05).

Additional subgroup analyses were performed according to sex and tooth side. No statistically significant differences in the prevalence of the evaluated anatomical variations were observed between the examined subgroups, except where reported in the primary analyses. Formal interaction analyses and sensitivity analyses were not performed because of the limited number of positive cases and the retrospective cross‐sectional design.

## 4. Discussion

The present CBCT‐based investigation evaluated the prevalence of MMCs, second distal canals, and additional distal roots in mandibular first molars and explored the potential association among these anatomical variations in an Iranian population. The principal findings demonstrated that MMCs were present in 13.0% of the evaluated teeth, second distal canals were identified in 32.0%, and additional distal roots were observed in 7.5% of cases. More importantly, statistically significant associations were identified between the presence of MMCs and distal anatomical complexity, including both second distal canals and additional distal roots. These findings suggest that anatomical variations within mandibular first molars may not occur as isolated phenomena but may instead represent manifestations of a broader pattern of developmental complexity affecting the entire root canal system [15–19, 29–32].

A fundamental objective of contemporary endodontics is the identification and management of the complete root canal system. Missed canals remain among the most frequently reported causes of persistent apical disease and posttreatment endodontic failure [12, 13, 22]. Consequently, understanding the prevalence and interrelationships of anatomical variations is of considerable clinical importance. While previous investigations have extensively described the occurrence of MMCs and distal root canal variations independently, relatively few studies have attempted to evaluate whether these anatomical structures coexist within the same tooth. The present study therefore contributes to a growing body of literature that seeks to move beyond simple prevalence reporting toward a more comprehensive understanding of anatomical interrelationships within mandibular first molars [14, 17, 19, 29].

The prevalence of MMCs observed in the present study (13.0%) falls within the broad range reported in previous CBCT investigations [15–20]. The considerable variability reported across studies is not unexpected and likely reflects differences in imaging modalities, voxel sizes, age distributions, ethnic backgrounds, sample selection strategies, and diagnostic criteria. Studies utilizing micro‐computed tomography frequently report higher prevalence rates than CBCT‐based investigations because of their superior spatial resolution and ability to identify very small accessory canals [14, 33–35]. Conversely, investigations relying on conventional radiography generally report substantially lower prevalence values due to the inherent limitations of two‐dimensional imaging. Therefore, direct comparison among studies should be interpreted cautiously.

The prevalence of second distal canals observed in the present study (32.0%) is generally consistent with previous reports from Middle Eastern and Asian populations [8, 15, 16, 24, 25]. The distal root anatomy of mandibular first molars has historically been considered less complex than the mesial root anatomy; however, accumulating evidence demonstrates that additional distal canals occur more frequently than previously recognized [8, 24, 25, 29]. The present findings support this growing consensus and reinforce the importance of careful exploration of the distal root system during endodontic treatment.

Similarly, the prevalence of additional distal roots identified in this study (7.5%) aligns with reports demonstrating considerable ethnic variability in the occurrence of radix entomolaris [8, 24, 25]. Previous investigations have shown that additional distal roots are particularly prevalent in East Asian populations, whereas lower frequencies are generally reported in European and Middle Eastern populations [8, 24, 25]. These differences have often been attributed to genetic and developmental influences, although the precise mechanisms remain incompletely understood. The prevalence observed in the present Iranian cohort appears intermediate when compared with values reported in East Asian and Western populations, supporting the notion that root morphology is influenced by population‐specific factors.

One of the most important findings of the present study was the significant association between MMCs and the second distal canals. Teeth exhibiting MMCs demonstrated significantly greater odds of presenting additional distal canal anatomy compared with teeth lacking MMCs. This observation suggests that increased anatomical complexity in one root may be accompanied by increased complexity elsewhere within the same tooth [17, 19, 36]. Although previous studies have occasionally reported similar tendencies, the evidence supporting these associations remains limited. Therefore, the present findings provide additional support for the concept that the root canal morphology should be viewed as an integrated developmental system rather than as a collection of independent anatomical structures.

The association between MMCs and additional distal roots should be interpreted particularly carefully. Although teeth exhibiting an MMC showed higher estimated odds of an additional distal root (adjusted OR = 6.00), the corresponding 95% CI was wide (1.33–27.16), reflecting the relatively small number of positive cases and limited statistical precision. Therefore, the magnitude of this association should not be interpreted as a precise estimate of the effect. Rather, the finding provides preliminary evidence of a possible relationship between mesial canal complexity and additional distal root anatomy that requires confirmation in larger datasets.

Importantly, no formal a priori sample size calculation was performed because the study retrospectively included all eligible CBCT examinations available during the predefined study period. Although this approach maximized the use of the accessible dataset, the resulting number of cases with uncommon anatomical variants was limited. Consequently, the study may have had insufficient statistical power to estimate associations involving rare anatomical features with high precision. Larger multicenter studies with prospectively defined sample size requirements are therefore needed to provide more stable effect estimates and narrower CIs.

The biological plausibility of these associations deserves particular consideration. Root formation is orchestrated through a highly regulated sequence of developmental events involving epithelial‐mesenchymal interactions, molecular signaling pathways, and morphogenetic regulation. Hertwig’s epithelial root sheath plays a central role in determining root number, root shape, and canal configuration by directing dentin deposition and root elongation [26, 28, 37, 38]. Minor variations during these developmental processes may influence the pattern of root bifurcation, canal partitioning, and accessory canal formation. Consequently, teeth exhibiting increased canal differentiation within the mesial root may possess a generalized developmental predisposition toward greater anatomical complexity throughout the entire root system.

This developmental interpretation is consistent with the observations reported in other areas of dental anatomy. Previous investigations have demonstrated that complex canal systems often coexist with additional roots, accessory canals, apical deltas, and isthmus formations [14, 29, 33, 34, 36]. Such findings suggest that anatomical complexity may represent a continuum rather than isolated morphological events. From this perspective, the coexistence of MMCs and distal anatomical variations observed in the present study may reflect a common developmental origin rather than independent anatomical occurrences.

Nevertheless, it is important to emphasize that the associations identified in this study should not be interpreted as predictive rules. The cross‐sectional design permits the evaluation of coexistence and association but cannot establish causality. Moreover, the presence of an MMC does not guarantee the existence of a second distal canal or additional distal root, nor does the absence of an MMC exclude distal anatomical complexity. Therefore, clinicians should regard these findings as indicators of increased probability rather than definitive predictors of root canal morphology.

The clinical significance of the present findings warrants careful consideration. Several previous studies have suggested that awareness of anatomical correlations may improve diagnostic vigilance and encourage a more comprehensive exploration of the pulp chamber floor [12, 13, 22]. In practical terms, the identification of an MMC during endodontic treatment may prompt clinicians to perform a more detailed assessment of distal root anatomy, particularly when advanced imaging or magnification systems are available. However, the utility of such associations should not be overstated. CBCT imaging itself remains capable of directly visualizing many anatomical variations, and clinical decision‐making should continue to rely primarily on careful examination and imaging findings rather than probabilistic anatomical assumptions.

The present findings should also be interpreted within the context of contemporary CBCT‐based anatomical research. Recent systematic reviews and multinational CBCT studies have emphasized substantial geographic and demographic variability in root canal morphology [15–17, 19, 20, 25]. The prevalence values reported in the present study contribute important data from an Iranian population and further highlight the necessity of population‐specific anatomical investigations. Such studies remain clinically relevant because endodontic treatment is ultimately performed within local populations whose anatomical characteristics may differ significantly from those reported elsewhere.

Despite the valuable information provided by CBCT, several methodological limitations inherent to this imaging modality must be acknowledged. Although CBCT offers excellent three‐dimensional visualization of dental structures and represents the most practical in vivo method for anatomical assessment, its spatial resolution remains substantially lower than that of micro‐computed tomography. Consequently, very small accessory canals, fine canal communication, isthmuses, and confluent canal systems may remain undetected. Recent comparative investigations have demonstrated that CBCT may underestimate the prevalence of complex canal anatomy when compared with micro‐CT [23, 33–35]. Therefore, the true prevalence of MMCs and other anatomical variations may be higher than that reported in the present study.

The issue of diagnostic sensitivity is particularly relevant for MMC detection. Many MMCs possess small diameters and frequently merge with either the mesiobuccal or mesiolingual canal in the apical third of the root [14, 18, 33, 36]. Such configurations may be difficult to distinguish from isthmuses or canal fins, particularly when larger voxel sizes are employed. Although standardized diagnostic criteria were applied in the present investigation, some degree of underdetection cannot be excluded. Future studies utilizing smaller voxel sizes or complementary micro‐CT analyses may provide additional insights into the prevalence and morphology of these structures.

Several additional limitations should be considered when interpreting the results. First, the retrospective cross‐sectional design precludes the establishment of causal relationships and limits the ability to evaluate developmental mechanisms directly. Second, the study was conducted within a single institutional setting, which may restrict the generalizability of the findings to other populations. Third, the relatively small number of MMC‐positive and additional distal root cases resulted in wide CIs, particularly in regression analyses. Consequently, although statistically significant associations were identified, the precision of some estimates remains limited. Fourth, a detailed evaluation of canal configuration patterns, isthmus morphology, root curvature, and canal merging behavior was beyond the scope of the present study. These anatomical characteristics may further contribute to understanding the developmental relationships among root canal variations and should be investigated in future research.

Notwithstanding these limitations, the present study possesses several strengths. The use of CBCT enabled a three‐dimensional assessment of root canal anatomy, reducing many of the limitations associated with conventional radiography [21, 22]. Standardized diagnostic criteria and examiner calibration procedures enhanced methodological reliability, while multivariable regression analyses allowed for adjustment for potential confounding variables. Furthermore, by focusing on anatomical associations rather than solely prevalence estimation, the study addresses a relatively underexplored aspect of root canal morphology and provides new insights into the potential interrelationships among anatomical variations within the mandibular first molars.

Future investigations should seek to validate these findings using larger multicenter datasets representing diverse ethnic and geographic populations. Prospective studies incorporating high‐resolution CBCT protocols and micro‐CT validation would further strengthen the understanding of anatomical complexity and developmental relationships [33–35]. Additionally, integration of genetic, anthropological, and developmental biology approaches may help clarify the mechanisms underlying the coexistence of multiple anatomical variations within individual teeth.

In summary, the present findings suggest that mandibular first molars exhibiting MMCs are more likely to demonstrate additional distal anatomical complexity, including second distal canals and additional distal roots. These observations support the concept that the root canal morphology represents an integrated developmental system in which variations may coexist throughout the tooth rather than occurring independently. While the clinical implications should be interpreted cautiously, recognition of these anatomical relationships may contribute to a more comprehensive understanding of root canal morphology and encourage greater awareness of potential anatomical complexity during endodontic treatment.

## 5. Limitations

Several limitations should be considered when interpreting the findings of the present study. First, the retrospective cross‐sectional design precludes the establishment of causal relationships between the MMCs and distal anatomical variations. Consequently, the observed associations should be interpreted as evidence of coexistence rather than developmental or predictive relationships.

Second, the study was conducted at a single academic center using a population from a specific geographic region of Iran. Although the findings contribute valuable information regarding root canal morphology in this population, caution should be exercised when extrapolating the results to other ethnic or demographic groups. Given the well‐documented influence of population‐specific factors on root and canal anatomy, multicenter investigations involving broader and more diverse populations are warranted.

Third, despite the inclusion of all eligible CBCT examinations available during the study period, the number of positive cases for certain anatomical variations remained relatively small. In particular, only a limited number of teeth exhibited MMCs and additional distal roots. As a result, some regression estimates were associated with relatively wide CIs, indicating reduced statistical precision and emphasizing the need for cautious interpretation of effect sizes.

Fourth, although CBCT is currently one of the most valuable noninvasive imaging modalities for in vivo assessment of root canal anatomy, its spatial resolution remains inferior to that of micro‐computed tomography. Consequently, very small accessory canals, canal communication, isthmuses, and confluent canal systems may not have been fully visualized. Therefore, the actual prevalence of complex anatomical structures may have been underestimated.

Fifth, the present investigation focused primarily on the presence or absence of MMCs, second distal canals, and additional distal roots. Other important anatomical characteristics, including canal configuration patterns, canal merging behavior, root curvature, apical anatomy, and isthmus morphology, were not comprehensively evaluated. These factors may provide additional insights into the developmental relationships among root canal variations and should be explored in future studies.

Finally, although examiner calibration procedures demonstrated substantial agreement, image interpretation remains inherently dependent on observer judgment. As with all radiographic studies, some degree of diagnostic variability cannot be completely eliminated.

Future research incorporating larger multicenter datasets, standardized CBCT acquisition protocols, detailed anatomical classifications, and micro‐CT validation is necessary to further elucidate the developmental and clinical significance of the associations identified in the present study.

## 6. Conclusion

Within the limitations of this retrospective CBCT‐based study, MMCs were associated with increased odds of second distal canals and additional distal roots in mandibular first molars. However, the relatively small number of positive cases, particularly for additional distal roots, resulted in wide CIs and limited the precision of the estimated associations. Therefore, these findings should be interpreted as preliminary evidence of coexistence between mesial and distal anatomical complexity rather than as definitive predictive relationships.

From a clinical perspective, identification of an MMC may warrant careful assessment of the distal root canal anatomy; however, the presence of an MMC should not be considered a reliable standalone predictor of additional distal anatomy. Larger multicenter studies with adequate sample sizes and standardized CBCT protocols are needed to confirm the observed associations and provide more precise estimates of their magnitude.

## Author Contributions

**Tooba Aslanabadi**: conceptualization, methodology, investigation, formal analysis, data curation, writing – review and editing. **Parisa Jalali**: investigation, data curation, writing – original draft, writing – review and editing. **Tahmineh Razi**: methodology, validation, writing – review and editing. **Saeedeh Galledar**: validation, writing – review and editing. **Naghmeh Rahimi Darehchi**: investigation, data curation, writing – review and editing. **Saeed Rahimi**: supervision, conceptualization, methodology, validation, writing – review and editing.

## Funding

This study was financially supported by the Research Council of Tabriz University of Medical Sciences.

## Disclosure

All authors have read and approved the final version of the manuscript. The corresponding author had full access to all of the data in this study and takes complete responsibility for the integrity of the data and the accuracy of the data analysis.

## Ethics Statement

This retrospective cross‐sectional observational study was approved by the Ethics Committee of Tabriz University of Medical Sciences (Approval Number IR.TBZMED.REC.1401.525) and was conducted in accordance with the Declaration of Helsinki. Because previously acquired CBCT images were analyzed retrospectively, the requirement for informed consent was waived by the institutional review board.

## Conflicts of Interest

The authors declare no conflicts of interest.

## Data Availability

Access to the data of this study is possible upon request to the corresponding author.
